# Label-Free Fluorescence Spectroscopy for Detecting Key Biomolecules in Brain Tissue from a Mouse Model of Alzheimer’s Disease

**DOI:** 10.1038/s41598-017-02673-5

**Published:** 2017-06-01

**Authors:** Lingyan Shi, Luyao Lu, George Harvey, Thomas Harvey, Adrián Rodríguez-Contreras, Robert R. Alfano

**Affiliations:** 1 Institute for Ultrafast Spectroscopy and Lasers, the City College of the City University of New York, New York, NY USA; 2 Department of Biomedical Engineering, Wenzhou Medical University, Zhejiang, China; 3 Department of Biology, the City College of the City University of New York, New York, NY USA; 4 Department of Physics, the City College of the City University of New York, New York, NY USA; 5 Riverdale Country School, Bronx, NY USA; 6 Department of Chemistry, Columbia University, New York, NY USA

## Abstract

In this study, label-free fluorescence spectroscopy was used for the first time to determine spectral profiles of tryptophan, reduced nicotinamide adenine dinucleotide (NADH), and flavin denine dinucleotide (FAD) in fresh brain samples of a mouse model of Alzheimer’s disease (AD). Our results showed that the emission spectral profile levels of tryptophan and NADH were higher in AD samples than normal samples. The intensity ratio of tryptophan to NADH and the change rate of fluorescence intensity with respect to wavelength also increased in AD brain. These results yield an optical method for detecting early stage of AD by comparing spectral profiles of biomolecules.

## Introduction

Alzheimer’s disease (AD), a degenerative disorder that attacks neurons in the brain and leads to the loss of proper cognition, is the sixth leading cause of death in the United States, and from 2000–2010 the proportion of deaths resulting from AD in America has gone up 68%^1^. Although AD has been the focus of much scientific research in past years, there is still no cure or understanding of molecular mechanisms. A large proportion of people with AD remained undiagnosed; early diagnosis can help them make decisions for the future while they are still capable, and can allow people to receive early treatment to improve their cognition and increase the quality of their life as they live with AD^2^.

Physicians diagnose AD with just an examination of the patients state, inquiries into the familial history of psychiatric and neurological disorders, and a neurological exam^1^. Other newer methods of diagnosis include using Magnetic Resonance Imaging (MRI) to look for Hippocampal atrophy^3^, Positron Emission Tomography (PET) scans^4^, and examining levels of beta-amyloid and tau protein in cerebrospinal fluids taken from the patient^5^. Photonics offers a new and novel approach to give molecular information on AD. In 1984, Alfano’s group pioneered the use of optical label free optical spectroscopy to detect cancer on a molecular level by looking at the fluorescence levels of native organic biomolecules in tissues^6^. This process of biomedical spectroscopy, using light and the native fluorescence of certain proteins and molecules within human tissue, has been expanded upon and applied to examine levels of tryptophan, reduced nicotinamide adenine dinucleotide (NADH), flavin, and collagen in normal and cancerous breast tissue for diagnosing certain types of cancer^7, 8^. The brain tissue is a smart tissue with different molecular components and structures in comparison to other body tissues. This past photonics work inspires the application of label free optical spectroscopy to detect AD at molecular level in the brain.

Mitochondria play an essential role in energy production by oxidative phosphorylation, and cell survival and death^9, 10^. Mitochondrial dysfunction has been associated to a number of diseases including cancer and AD^10–12^. Early identification of mitochondrial dysfunction will be helpful for early detection and better understanding the mechanisms of AD. Intracellular coenzymes such as NADH and flavin adenine dinucleotide (FAD) play important roles in cellular oxidation-reduction (redox) reactions^9^, thus can be potentially used as intrinsic biomarkers for detecting metabolic activities and mitochondrial dysfunction. Change of NADH-linked mitochondrial enzymes has been found in AD brain^13, 14^. Tryptophan kynurenine metabolism has also been reported involved in the pathogenesis of AD^15^.

In the present study we measured the fluorescence spectroscopy in mouse brain tissue with an early stage of AD^16^, and in normal brain samples for comparison purpose. The objective was to develop a technique that applies biomolecules (tryptophan, NADH, and FAD) as intrinsic biomarkers for detecting early stage of AD in mouse brain tissue, and to propose a potential method for detection, diagnosis, and better understanding of AD in humans.

## Results

**Figure 1:**
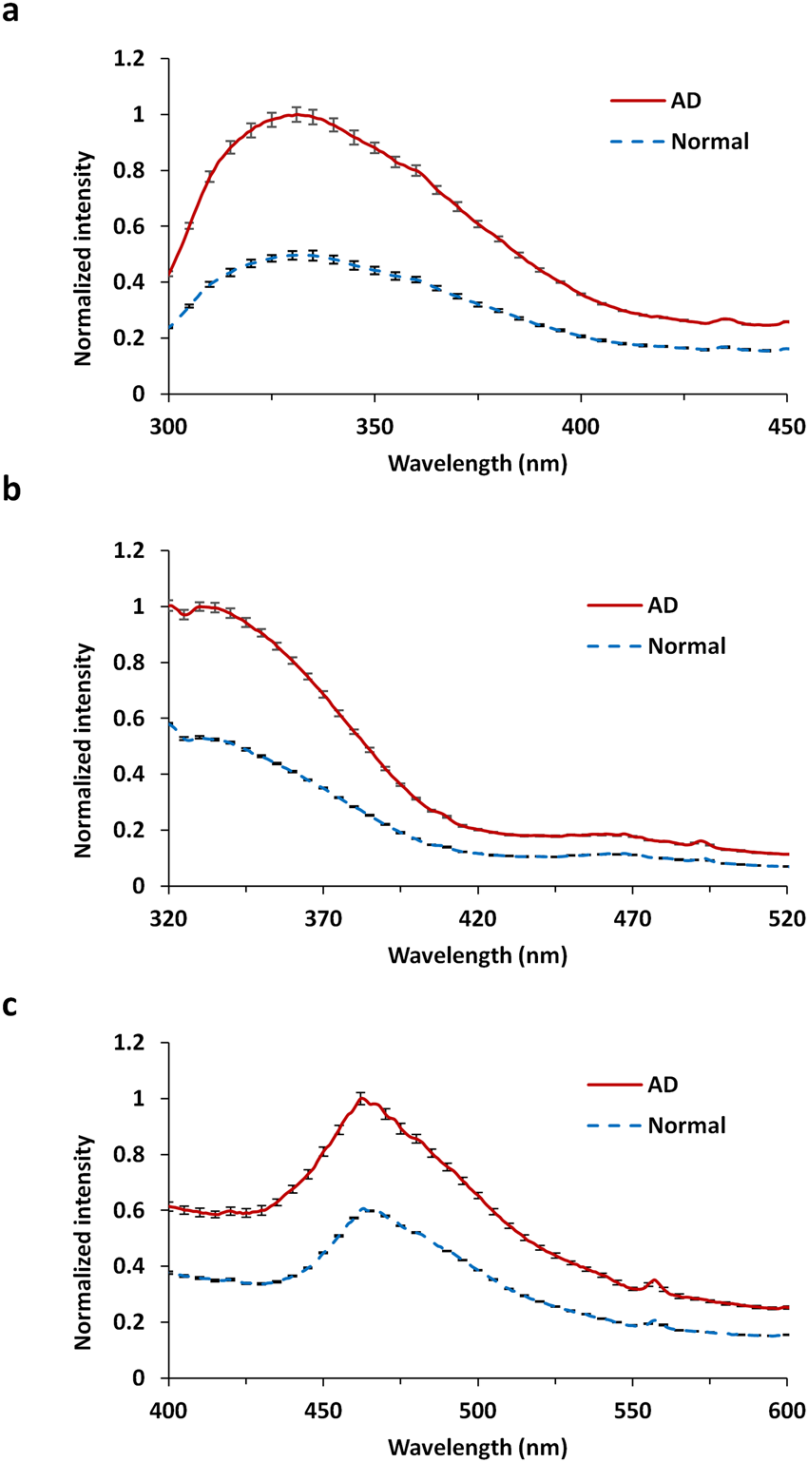
Averaged spectral (emission) profiles with error bar (n = 5) of AD and N brains at excitation wavelength (**a**) 266 nm, (**b**) 300 nm, and (**c**) 340 nm.

**Figure 2:**
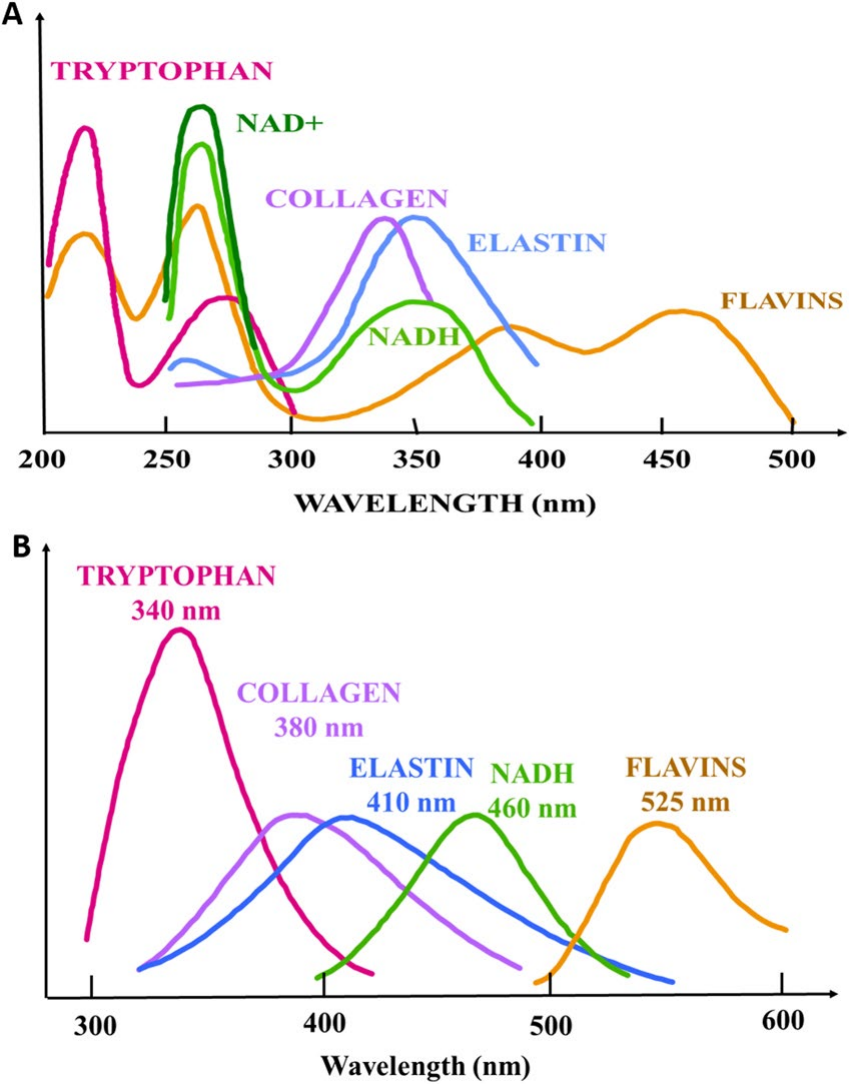
Absorption and fluorescence profiles of key biomolecules. (**a**) Absorption of key molecules, and (**b**) emission of key molecules.

Figure 1 displays the result of fluorescence spectral profiles in AD and N brain samples at excitation wavelengths 266 nm (Fig. 1a), 300 nm (Fig. 1b), and 340 nm (Fig. 1c). Different excitation wavelengths were employed to determine the emission spectra of each biomolecule (tryptophan, NADH, and FAD), as shown in Fig. 2. Table 1 summarizes the emission wavelengths for assigned molecules at peak emissions in AD and N fresh brain tissues under different excitation wavelengths.

**Table 1:** Emission peaks in AD and N brain samples.

Excitation wavelength	Tissue	Normalized intensity of peak 1	Normalized intensity of peak 2	Ratio (peak1/ peak2)
266 nm		tryptophan emission at 331 nm	NADH emission at 435 nm	
	AD	1.032	0.266	3.88
		1.016	0.271	3.75
		1.001	0.269	3.73
		0.986	0.268	3.68
		0.965	0.268	3.60
	mean	1.000	0.268	3.73
	N	0.522	0.174	3.01
		0.495	0.170	2.91
		0.495	0.169	2.93
		0.491	0.167	2.93
		0.480	0.167	2.88
	mean	0.497	0.169	2.93
		tryptophan emission at 335 nm	NADH emission at 492 nm	
300 nm	AD	1.013	0.164	6.19
		1.014	0.161	6.29
		1.005	0.161	6.25
		0.989	0.161	6.15
		0.979	0.158	6.19
	mean	1.000	0.161	6.21
	N	0.536	0.101	5.31
		0.537	0.100	5.36
		0.531	0.099	5.35
		0.530	0.099	5.34
		0.526	0.099	5.31
	mean	0.532	0.100	5.33
		NADH emission at 462 nm	FAD emission at 557 nm	
340 nm	AD	1.032	0.358	2.88
		1.010	0.355	2.84
		0.998	0.353	2.83
		0.983	0.348	2.82
		0.978	0.343	2.85
	mean	1.000	0.352	2.84
	N	0.606	0.212	2.86
		0.609	0.206	2.95
		0.609	0.205	2.97
		0.605	0.206	2.93
		0.602	0.207	2.92
	mean	0.606	0.207	2.928

AD: Alzheimer; N: normal.

Figure 1(a) shows that at excitation wavelength of 266 nm the fluorescence peaks of AD and N brain tissues are at the same wavelength (~330 nm), corresponding to the wavelength of emission peaks of tryptophan (Fig. 2)^17^. Significant difference of peaks of tryptophan was observed between AD and N brain (P = 0.001). A weak secondary peak ranging from 430 to 460 nm is due to NADH, which may be caused by fluorescent resonance energy transfer from excited tryptophan to NADH and second singlet excitation from 266 nm. The averages of the two peak intensities in AD brain are 2.01-fold (for tryptophan, 1.000 vs. 0.497) and 1.58-fold (for NADH, 0.268 vs. 0.169) higher, respectively, than those in N brain (Table 1).

Figure 1(b) shows the scans at excitation wavelength of 300 nm, which are similar with emission spectra excited at 266 nm. The emission intensities of the AD and N brain tissues both peak in the range of 330–350 nm, which match the wavelength of the emission peak of tryptophan (Fig. 2). In addition, the weak second peaks are at 430–460 nm due to NADH. The mean peak intensity of tryptophan and NADH in AD brain tissue are 1.88-fold (1.000 vs. 0.532) and 1.61-fold (0.161 vs. 0.100) higher, respectively, than those in N brain tissue (Table 1).

Figure 1(c) shows the scans at excitation wavelength of 340 nm. The emission peaks of AD and N in the range of 430–460 nm match the emission peak of NADH (Fig. 2), and the weak second peaks at 530–560 nm is due to FAD. The mean peak intensities of NADH and FAD are 1.65-fold (1.000 vs. 0.606) and 1.70-fold (0.352 vs. 0.207) higher, respectively, in AD brain compared to N brain (Table 1).

**Figure 3:**
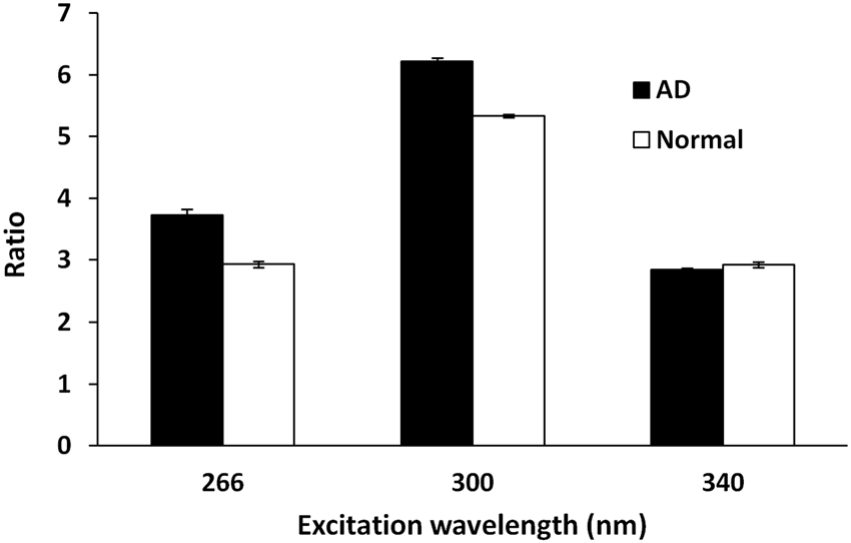
Ratios of intensity peaks from three different regions of interest. Ratios of emission peaks (**a**) of tryptophan/NADH (at 331 nm/435 nm) when excited at 266 nm, (**b**) of tryptophan/NADH (at 330 nm/490 nm) when excited at 300 nm, and (**c**) of NADH/FAD (at 462 nm/557 nm) when excited at 340 nm.

An alternate way to differentiate the spectral profiles in AD or N brain is to compare the intensity ratio of tryptophan to NADH (Table 1, Fig. 3). The average values of the ratio are 3.73 in AD brain and 2.93 in N brain at the excitation wavelength of 266 nm, and 6.21 in AD and 5.33 in N excited at the wavelength of 300 nm. The increased ratio of tryptophan to NADH in AD indicates low efficiency of energy transfer from tryptophan (donor) to NADH (acceptor) which may be due to longer distance (R) and fewer interactions between the two molecules. Comparing the spectral profiles (peaks) of tryptophan and NADH and their relative ratio excited at the wavelength near absorption peak of tryptophan may be an applicable method for diagnosing AD. On the other hand, as shown in Fig. 3, the ratios of NADH to FAD in AD brain are not significantly different from the ratios in N brain, which indicates that analogous changes of NADH and FAD occurred in AD brain.

**Figure 4:**
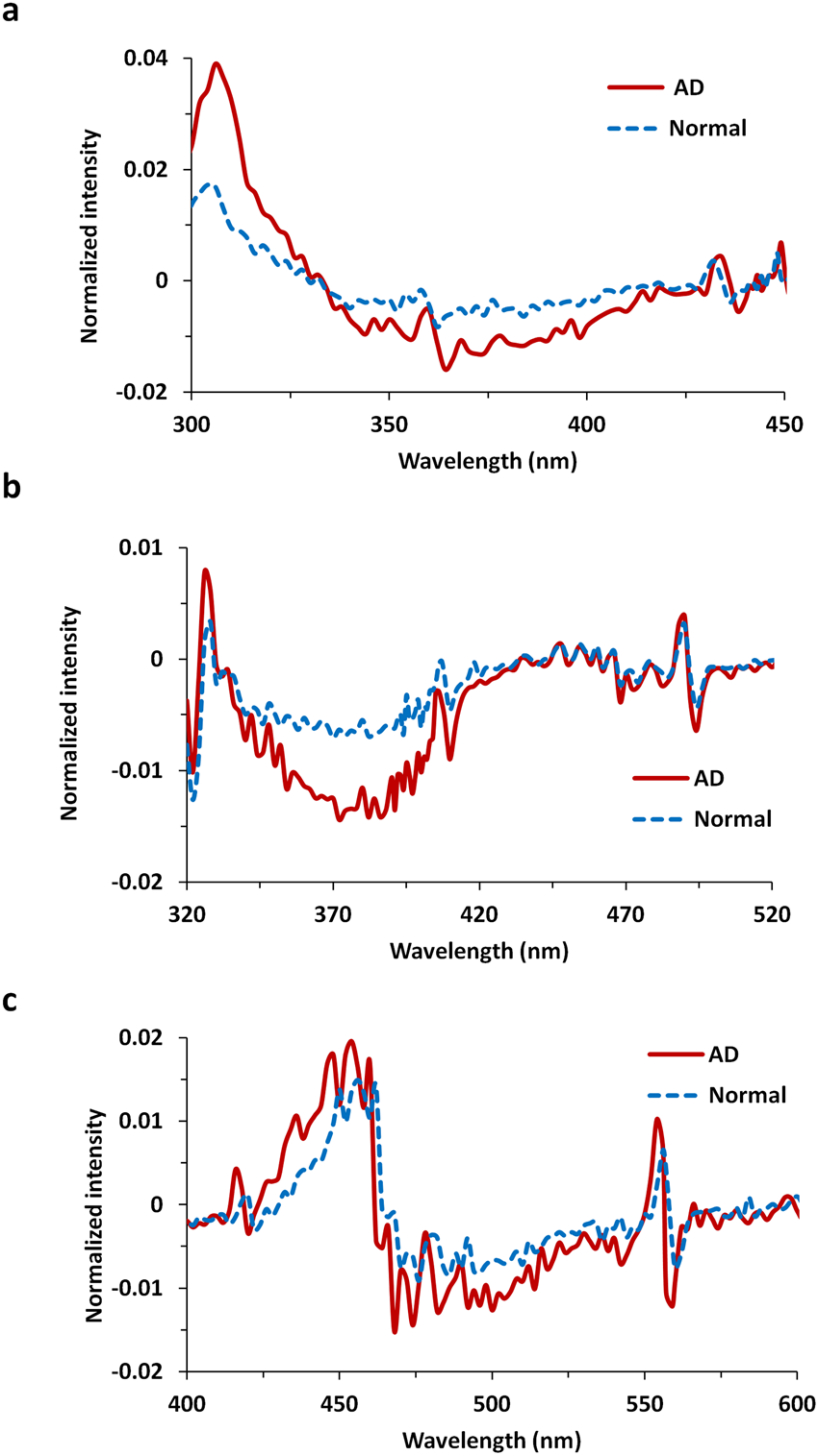
Means of the first derivative of fluorescence profiles of AD and N brain tissues at excitation wavelength (**a**) 266 nm, (**b**) 300 nm, and (**c**) 340 nm.

The first derivatives of emission spectra were calculated for comparing fluorescence properties of AD and N brain tissues. Figure 4a–c show the mean profiles of the first derivative of emission spectra which were excited by monochromatic excitation lights of 266 nm, 300 nm, and 340 nm, respectively. At excitation wavelength of 266 nm, the ascending rate of emission intensity is higher in AD brain than that in N brain, the peaks of which are 0.039 vs. 0.0169; and the descending rate of intensity in AD is higher than that in N brain, the negative peaks of which are about −0.0159 vs. −0.0083. When excited at 300 nm, the maximum values of the ascending rate are 0.0078 in AD and 0.0034 in N brain; and the negative peaks of the descending rate are −0.0144 in AD and −0.0071 in N brain. However, the derivative of spectra from AD brain is close to that from N brain at excitation wavelength of 340 nm (Fig. 4c), due to the similar curve shapes of fluorescence spectra in both AD and N brain tissues (Fig. 1c). The derivative of spectral profiles could be used to measure instantaneous rate of change, the ratio of the instantaneous change in the fluorescence intensity to that of its wavelength.

## Discussion

In our experimental results, fluorescence intensities of tryptophan, NADH, and FAD were higher in the brain tissues of a young transgenic AD mouse compared with N brain tissues. The increase in emission intensity at about 340 nm of direct pumping tryptophan shows more emission efficiency in AD than N, which may be due to decreased nonradiative Knr or increased Kr. This is because tryptophan may be in a cage and has fewer interactions to the host molecules in the environment in AD than in N brain. This observation is consistent with the results from THz research in AD and N^18^. Therefore, the vast disparity of tryptophan fluorescence levels in AD and N mouse brain scans proposes an important method for AD diagnosis. Mitochondrial abnormalities are correlated with AD, while intracellular NADH and FAD play important roles in mitochondrial dysfunction that allows them as potential biomarkers for diagnosis of AD^9^, and this is validated by the current study. Nevertheless, NADH-linked mitochondrial enzyme activity was reported to be down-regulated in AD patients^13^, our results showed higher NADH emission efficiency. One reason might be the different host environment of biological molecules in AD, in which NADH is farther from tryptophan and NADH itself may also have fewer interaction with the host environment. As a result, the emission intensity of NADH was higher in AD due to reduced nonradiative Knr or increased radiative Kr. Considering our objective was to detect AD in its early stage such that we used a young AD mouse, another reason may be due to overcompensation of NADH for dysfunction of energy metabolism in the early stage of AD. The future direction could use time resolved fluorescence that gives fluorescence rate (K_f_ = Kr + Knr) and combines with longer wavelength multiphoton excitation that offers deeper tissue penetration.

In the present study, the scattering of fluorescence intensity is small since 1) the emission is detected from <0.5mm deep from the surface, and 2) the scattering coefficient and transport coefficient are smooth and flat, causing little or no influence on the measurements (as shown in Fig. 1).

In conclusion, the current study shows for the first time the fluorescence spectra of major molecular building blocks in brain of tryptophan, NADH, and FAD in AD and N mouse brain tissues. Fluorescence intensity levels of tryptophan, NADH, and FAD increased in AD brain tissues. This study verifies that tryptophan, NADH, and FAD can be employed as biomarkers for AD diagnosis. This work provides an effective technique to detect differences of fluorophore compositions in AD and normal brain tissues, and to diagnose AD by examining the spectral profiles of various fluorophores. This research can extend to employ ultrafast time resolved two photon excitation fluorescence spectroscopy for measuring the underlying relaxation times in AD.

## Methods

### Animal preparation

Mice were purchased from Jackson Laboratory and housed at the City College Animal Facility. A 3-month-old triple transgenic AD mice harboring PS1M146V, APPSwe and tauP301L transgenes in a uniform strain background^19^ was used. Another N mouse at the same age was used as control. The experimental methods were in accordance with the guidelines and regulations approved by the Institutional Animal Care and Use Committee at the City College of the City University of New York. The protocol number is 841. The method used to prepare rodent brain tissue has been described in detail elsewhere^18^. A brief outline of the methods is given below with emphasis on the special features of the present experiments.

After anesthesia with a mixture of ketamine and xylazine (41.7 and 2.5 mg/kg body weight, respectively), the mouse was decapitated and the brain was dissected and taken out. Fresh brain tissue with the hippocampus region was quickly sliced coronally at thickness of ~2 mm with a brain matrix (RWD Life Science Inc., CA). The fresh brain tissue slice was then immediately placed in a quartz cuvette. Regions of interest (ROI) in the hippocampus were measured 5 times at different spots in each AD and normal brain samples.

### Basic theory of fluorescence

It is well known that the fluorescence intensity I_f_ depends on efficiency Q from the radiative rate Kr and nonradiative rate Knr, where Q is given by^20^:

1 $${\rm{Q}}={\rm{Kr}}/({\rm{Kr}}+{\rm{Knr}})$$

where Q equals to the ratio of number of photons emitted out to the number of photons pumped in (Nout/Nin). The intensity from excited molecules I_f_ is

2 $${{\rm{I}}}_{{\rm{f}}}=({\rm{\Omega }}/4{\rm{\pi }})({\rm{Q}}\cdot {\bf{n}})$$

where Ω is the solid angle and **n** is the number of excited molecules. The Knr depends on the interaction of molecules with their host environments. Weak interaction will lead to a small Knr and more emission intensity. When Knr ≫ Kr, the emission is reduced.

Förster resonance energy transfer (FRET) is a mechanism for energy transfer between donor and acceptor via dipole-dipole coupling. Since the emission peak of tryptophan is around 340 nm and the absorption peak of NADH ranges from 340~360 nm, energy transfer from excited donor (tryptophan) to acceptor (NADH) probably occurs in the biological tissues^21, 22^. Effective donor to acceptor transfer can reduce emission from donor and enhance emission from acceptor. The transfer rate is

3 $${{\rm{K}}}_{{\rm{DA}}} \sim (1/{{\rm{\tau }}}_{{\rm{D}}}){({{\rm{R}}}_{0}/{\rm{R}})}^{6}$$

where R_0_ is overlap between donor emission and acceptor absorption, τ_D_ is the fluorescence lifetime of donor, and R is the distance between donor and acceptor.

### FluoroMax-3 fluorescence spectrometer

The fluorescence of AD and N brain tissues was measured by a FluoroMax-3 fluorescence spectrometer (Horiba Jobin Yvon Inc.). A 150-W xenon lamp was used as the discharge light source in the spectrometer. There are two Czerny-Turner monochromators for excitation and emission respectively. The essential part of a monochromator is a reflection grating, which selects the wavelength being used. The gratings contain 1200 grooves mm^−1^. A direct drive is used for each grating to scan the spectrum at up to 200 nm/s, the accuracy is better than 0.5 nm and repeatability is of 0.3 nm. The monochromatic excitation light strikes the sample, which is stored in a cuvette, and then emits fluorescence. The fluorescence light is directed into the emission monochromator, and is collected by the signal detector whose response ranges from 180–850 nm. Another detector named reference detector monitors the xenon lamp, and has good response from 190–980 nm.

The AD and N brain samples were excited at selected wavelengths 266 nm, 300 nm, and 340 nm, respectively, to examine the fluorescence peaks of each of tryptophan, NADH, and FAD. All measurements were performed by using a scanner (at 200 nm/sec), and the samples were held in cuvettes during the measurement.

Measurements of AD and N brain samples were each taken at three regions of interest, with the same slit width of 2.0 nm (in bandpass unit) and integration time of 0.2 s at each excitation wavelength. Three groups of spectra were obtained at excitation 266 nm, 300 nm, and 340 nm, respectively. Each group contains three spectra from AD brain tissues and three from N brain. Average curve of these three spectra and maximum intensity were calculated. In each group, the spectral profiles were normalized to the maximum intensity of averaged spectra from AD brain. All averaged data was presented as mean ± SD.
